# Coverage and Lifetime Optimization by Self-Optimizing Sensor Networks †

**DOI:** 10.3390/s23083930

**Published:** 2023-04-12

**Authors:** Franciszek Seredyński, Tomasz Kulpa, Rolf Hoffmann, Dominique Désérable

**Affiliations:** 1 Institute of Computer Science, Cardinal Stefan Wyszyński University, 01-938 Warsaw, Poland; 2 Department of Computer Science, Technische Universität Darmstadt, 64289 Darmstadt, Germany; 3 Institut National des Sciences Appliquées, 35700 Rennes, France

**Keywords:** collective behavior, network coverage and lifetime, second-order CA, self-optimizing networks, spatial prisoner’s dilemma, wireless sensor networks

## Abstract

We propose an approach to self-optimizing wireless sensor networks (WSNs) which are able to find, in a fully distributed way, a solution to a coverage and lifetime optimization problem. The proposed approach is based on three components: (a) a multi-agent, social-like interpreted system, where the modeling of agents, discrete space, and time is provided by a 2-dimensional second-order cellular automata, (b) the interaction between agents is described in terms of the spatial prisoner’s dilemma game, and (c) a local evolutionary mechanism of competition between agents exists. Nodes of a WSN graph created for a given deployment of WSN in the monitored area are considered agents of a multi-agent system that collectively make decisions to turn on or turn off their batteries. Agents are controlled by cellular automata (CA)-based players participating in a variant of the spatial prisoner’s dilemma iterated game. We propose for players participating in this game a local payoff function that incorporates issues of area coverage and sensors energy spending. Rewards obtained by agent players depend not only on their personal decisions but also on their neighbor’s decisions. Agents act in such a way to maximize their own rewards, which results in achieving by them a solution corresponding to the Nash equilibrium point. We show that the system is self-optimizing, i.e., can optimize in a distributed way global criteria related to WSN and not known for agents, provide a balance between requested coverage and spending energy, and result in expanding the WSN lifetime. The solutions proposed by the multi-agent system fulfill the Pareto optimality principles, and the desired quality of solutions can be controlled by user-defined parameters. The proposed approach is validated by a number of experimental results.

## 1. Introduction

WSNs have become today one of the key information and communication technologies [1] of the internet of things and a basic component of emerging intelligent services termed ambient intelligence. They are networks of a large number of tiny computer-communication devices called sensors which are deployed in some areas to sense a local environment. Their main task is collecting, sending, and processing a large amount of data necessary to provide intelligent services. In many applications, e.g., monitoring remote and difficult-to-access areas, sensors are equipped with single-use batteries that cannot be recharged.

From the quality of service (QoS) point of view of such WSNs, there exist two closely related important issues: the effective monitoring (coverage) of some areas and an operational lifetime. After the deployment (e.g., by an aircraft) of sensors at random locations, they should self-organize: recognize their nearest neighbors to be able to communicate and start taking local decisions in subsequent moments about turning on or off their batteries to monitor events. These decisions will directly influence the degree of area coverage, spending sensors’ batteries energy, and the lifetime of the network. The problem of lifetime maximization is closely related to the coverage problem. A group of sensors monitoring some area is usually redundant, i.e., usually more than one sensor covering monitored targets, and forms of redundancy can be different. By solving the coverage problem, one can also indirectly solve the WSN lifetime maximization.

There exists a number of algorithms to solve the problem of coverage/lifetime maximization. They are classified either as centralized, assuming the availability of the entire information and a solution is delivered usually in the form of a schedule of activities of all sensors during the entire lifetime, or distributed, where a solution is found based on only partial information about the network. Because these problems are known as NP-complete [2], WSN centralized algorithms are oriented either on the delivery of exact solutions for specific cases (see [3]) or applying heuristics or metaheuristics to find approximate solutions (see [4,5,6,7]). The main drawback of centralized algorithms is that a schedule of sensors’ activities must be found outside the network and delivered to it before starting operation. Therefore, distributed algorithms become more and more popular because they assume the reactivity of sensors in real time, and they are scalable in contrast to centralized algorithms.

In this paper, we present a novel approach to solve the problem of coverage/lifetime optimization in a fully distributed way by self-optimization. Self-optimization is a further extension of the concept of distributed algorithms, and it assumes that entities (agents) participating in searching for a solution act locally without knowledge of an optimized goal of the system. We present the results of a number of experimental studies showing different aspects of the behavior of the proposed algorithm. The paper is an extended version of [8], where we proposed a concept of self-optimizing WSN and presented initial experimental results confirming the capability of self-optimization of such networks. Our approach is based on applying the recently proposed [9] methodology called *Competitive Co-evolutionary CA-based System* extending our works [10,11], which assumes a multi-agent interpretation of a problem, and the competitive co-evolution of CA-based agents participating in the spatial prisoner’s dilemma (SPD) game, with the use of a second-order CA [12] as players. The paper also extends our works and concepts presented in [13,14], and  concerning the development of distributed algorithms, where learning automata and classical CA, respectively, were applied. The main contributions of the paper are as follows:

- An interpretation of a WSN as a multi-agent system represented by a WSN graph with agents representing sensors was proposed;
- A second-order CA-based agents of the multi-agents system making decisions concerning turning on/off their batteries was proposed;
- An interaction between agents is described by principles of a variant of the SPD game;
- A payoff function used by agents in the game, incorporating issues of area coverage and sensors energy spending was proposed;
- A number of experiments have been conducted to find the settings of the payoff function parameters;
- A number of experiments have been conducted to show that the system is able to self-optimize, i.e.,  to find near-optimal values of external performance criteria represented by the requested coverage and a minimal energy spending only due to local interactions between players, without their knowledge about the current values of the criteria;
- It was also shown that the solutions found by the system fulfill the Pareto optimality principles, and these solutions can be controlled by the corresponding settings of the payoff function parameters.

The structure of the paper is as given in the following. The next section presents a literature overview concerning centralized, distributed and self-optimizing algorithms of coverage and lifetime optimization problems in WSN and game-theoretical models related to our problem. Section 3 presents the problem of coverage/lifetime optimization in WSN. Section 4 contains a description of the concept of collective behavior of a second-order CA. A multi-agent system based on the concept of collective behavior of CA-based players in SPD game, solving WSN coverage and lifetime optimization problems by self-optimization, is presented in Section 5. Section 6 presents the results of the experimental study, and the last section contains conclusions and outlines future works.

## 2. Related Work

Lifetime maximization and, related to it, the coverage problem are usually considered variants of the scheduling problem, where it is necessary to make a sequence of decisions saying which subset of sensors should be turned on at a given moment of time to maintain a requested coverage of WSN with the use of a minimal number of active sensors. These problems are subjects of intensive studies, results of which can be found in the current literature (for an extensive overview, see [15]). A number of centralized and distributed algorithms have been proposed recently to solve these problems. Centralized algorithms can be further classified as algorithms providing either exact or approximate solutions.

**Centralized algorithms.** The covering problem is computationally expensive, and exact solutions can be found only for relatively small instances of the problem with the use of classical optimization algorithms, such as linear programming [16] or integer programming [17]. For realistic instance sizes of the problem, one can rely on applying some heuristic (see [18]) or metaheuristic, which can deliver approximate, near-optimal solutions.

Currently, one of the most popular techniques to solve the coverage problem are ones based on applying nature-inspired metaheuristics. They belong to the class of centralized algorithms and require full information about the state of the system. To solve these problems, different versions of evolutionary algorithms were applied. The most popular evolutionary techniques are genetic algorithms (GA) (see [19]). They were used recently by [4] to solve the target coverage problem, and by [20] for lifetime optimization by applying a method combining mixed integer programming with GA. In [21], the authors proposed an energy-efficient coverage method, where GA is used to compute the minimum number of potential positions to allow all targets to be *k*-covered and all sensor nodes to be *m*-connected. In [22], a hybrid approach was proposed, where, in particular, GA was used to generate a connected graph that provides a minimal path that links deployed sensors and maintains coverage.

A recent paper [7] applies a novel approach called a *hyperheuristic*, where an evolutionary technique—genetic programming, which is a high-level heuristic—is used to solve the problem created on the base of a set of low-level heuristics. A variant of the evolutionary technique called memetic algorithms [23] was used to solve the coverage problem. Another popular technique is particle swarm optimization. It was applied to solve the coverage problem considered either as a single objective problem [24,25,26] or a multi-objective problem [27].

Recently a number of novel metaheuristics were applied to solve problems related to WSN. In [28], the artificial bee colony algorithm has been proposed to solve the WSN coverage and connectivity problem. In [29], the metaheuristic called bacterial foraging optimization scheme was applied to maintain coverage, energy efficiency, and a high network availability in mobile-sensing WSN. Two other optimization techniques, such as harmony search [30] and ant colonies, were also recently applied [6].

**Distributed algorithms.** A distributed algorithm assumes that there exist separate autonomous parts of an algorithm that can run simultaneously on independent processors and use limited information about other parts. The main problem related to distributed algorithms is the need for coordinating the behavior of independent parts of the algorithm to achieve a consensus about how to reach a global goal. Solving this problem requires some level of global communication between all independent parts, which may influence the decreasing performance of the algorithm.

During recent years, we have been able to observe an increasing interest in designing distributed algorithms to solve the coverage/lifetime maximization problems in WSN with the use of learning automata (LA) [31,32]. LA are a class of reinforcement learning machine algorithms that can be directly used to solve learning and optimization problems. These algorithms have a number of advantages in comparison with centralized algorithms. They consider a given problem as a distributed system consisting of a number of autonomous agents and focus on the description of local dependencies between agents and their actions to solve collectively a problem. LA were applied to solve different variants of the coverage/lifetime maximization problems in WSNs. In particular, in [33], authors proposed a scheduling method based on LA, in which each sensor is equipped with a learning automaton that decides to select either a state to be active or to sleep in some moments of time. Authors in [34] considered the problem of lifetime maximization with the use of LA. To prolong the network’s lifetime, the authors in [35] proposed a heuristic algorithm based on applying LA to schedule sensors’ activity to minimize the energy spending for the target coverage problem. In [36], cellular learning automata have been proposed for the minimization of energy consumption in WSN and increasing the lifetime through the formation of groups.

The main problem that remains here with LA is the way to a solution of the consensus problem related to an optimization goal, e.g. the level of coverage. This goal is global and requires some central instance, collecting information from all agents about their local state to calculate the global level of coverage, before informing them back about their rewards or penalties. This requires the existence of a special node with large computing power and communication costs, that limits the application of this approach in large real-time WSN systems. This problem can be solved by using self-optimizing algorithms.

**Self-optimizing algorithms.** Self-optimizing algorithms can be considered as a specific class of distributed algorithms where separate autonomous parts of an algorithm can run simultaneously on independent processors. They use limited information about the other parts but they can adapt in time in an environment by a non global, local-only communication to solve a consensus problem. The issue of working out self-optimization algorithms [37] has recently been recognized in the context of the grid and ubiquitous computing systems [38], mobile communication systems [39], or load balancing in distributed systems [40]. To our knowledge, self-optimizing algorithms to solve the coverage/lifetime maximization problems in WSN are unknown in the current literature.

To design self-optimizing algorithms both LA and CA [41] can be used. One possible way is the requirement to interpret the problem in terms of a multi-agent system where interactions between agents are described in terms of non-cooperative games [42], and to solve the problem of global consensus by designing an appropriate payoff function that incorporates area coverage issues and sensors energy spending, usually formulated as a global optimization goal.

Our first attempt in this direction was presented in [13]. A game-theoretic model related to the problem of lifetime optimization in WSN with the use of deterministic ϵ-LA as players in the games was presented together with a payoff function reflecting the issues of the WSN coverage and lifetime. While the first experimental results were promising, we noticed that the payoff function needs to be improved. Further work was continued with the use of CA.

Classical CA are not learning machines, and applying them directly to solve optimization problems requires some effort, i.e., requires discovering CA rules which are able to solve a problem. The study presented in [43] is promising and shows that there exists a strong relation between levels of coverage and lifetime of WSN, and specific CA rules describing the behavior of CA-based agents controlling and activating the nodes’ batteries. Our recent work [14] shows the potential of such an approach. In this context, it is also worth noticing the work [44] where a CA-based approach to the observability problem was defined in the context of an autonomous network of mobile sensors considered a control theory system, and oriented on an ability to reconstruct the initial system’s state.

One possible way to provide CA with learning and optimization capabilities is to convert them into a second-order CA, where a mechanism of evolutionary competition enables the changing of the assigned CA rules during their evolution, as it was proposed in [10,11], and consider them reinforcement learning machines operating in an SPD environment, or combining the CA model with the spatial–temporal evolutionary process and learning automata theory like it was proposed in [45]. Our recent study [8] shows that the idea of applying a second-order CA to solve the coverage and lifetime optimization problem together with developing a new payoff function reflecting issues of WSN coverage and sensors energy spending is very promising, and in this paper, we will study it in detail.

In this context, it is also worth noticing the works [46,47]. In [46], the authors combine 2D CA cells with concepts of GA, allowing evolving CA cell rules due to local mating into a desired direction. The authors of [47] combine game theory concepts with GA concepts, which results in designing very effective GA operators.

## 3. Coverage and Lifetime Problems in Wireless Sensor Networks

**Figure 1:**
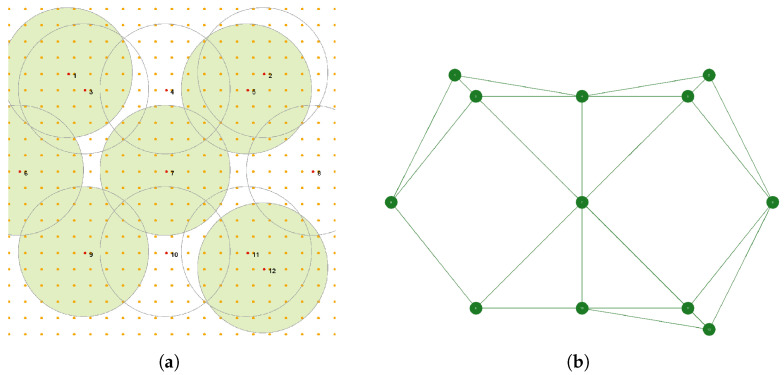
Example of a monitored area containing 441 PoI with WSN 12 consisting of 12 sensors with $R_{s}=20$ m (**a**); corresponding WSN graph for WSN 12 (**b**).

We assume that some area of the size of $L_{1}$×  $L_{2}$ m ${}^{2}$ should be monitored by a sensor network $S=\{s_{1},s_{2},\cdots ,s_{N}\}$ consisting of *N* sensors randomly deployed over this area. More specifically, the area is represented by *M* “points of interest” (PoI), which regularly cover the area and should be monitored. Each sensor has a non-rechargeable battery of capacity batt_capacity and can monitor PoI in a sensing range $R_{s}$ if its battery is turned on. An energy capacity of a sensor is decreased by one unit of energy per single interval of time if the battery is turned on. Figure 1a shows an example of such an area with $L_{1}$ = $L_{2}$ = 100 m with M=441 PoI (in orange), N=12 sensors, and $R_{s}=20$ m; some of the sensors are currently turned on and monitor the corresponding areas (in green).

It is assumed that decisions about turning on/off the batteries are taken in discrete moments of time *t*. It is also assumed that there exists some QoS measure evaluating the performance of the WSN. As such a measure, we accept a value of coverage defined as the ratio of the number of PoI covered by active sensors to the whole number *M* of PoI. The coverage *q* of a target area can be denoted as

(1) $$q_{j}=\frac{M_{obs_{j}}}{M}$$

at the *j*-th time period $t_{j}$.

A desirable objective is to preserve the complete area coverage, but sometimes it may be more practical to achieve a predefined coverage rate that is just high enough. So, we assume that at a given moment, this ratio should not be lower than some predefined requested value $q_{r}$$\left(0<q_{r}\leq 1\right)$. The lifetime of WSN can be defined as the number of time intervals $t_{j}$ in the schedule, during which the coverage of the target area is within the range δ of the requested coverage ratio $q_{r}$, as follows:

(2) $${\mathrm{lifetime}}_{q_{r}}=\sum_{j=1}^{T_{max}}t_{j},$$

where $t_{j}=1$ if $abs(q_{j}-q_{r})\leq \delta$, otherwise $t_{j}=0$.

The objective is to prolong the lifetime of WSN by minimizing the number of redundant sensors during each time interval to minimize energy consumption, providing at the same time the requested value of coverage. One can notice that a crucial component of this objective is solving the coverage problem, i.e., finding a minimal number of sensors that cover the monitored area with the requested coverage ratio $q_{r}$. In this paper, we will focus on solving this component of our objective.

To solve our problem and deliver a distributed algorithm of a solution, we will use a multi-agent approach. For this purpose, we will assume that each sensor $s_{i}$ of WSN is controlled by an agent $A_{i}$ of a multi-agent system consisting of *N* agents. To represent our problem by a multi-agent system, we need to convert a WSN deployed in some area into a WSN graph (see, the next section) as it is shown in Figure 1b. Interactions between agents will be described by the principles of an iterated SPD game and the emerging collective behavior in such a game.

## 4. Collective Behavior of the Second-Order CA-Based Players in Spatial Prisoner’s Dilemma Game

Recently [10,11], we showed that second-order CA participating in SPD games are able to self-organize, i.e., as a result of the collective behavior, they can maximize a global criterion related to the system that is unknown for the agent players. Below, we briefly present the key issues related to SPD games.

We consider a 2D spatial array of size m×n. We assume that a cell (i,j) will be considered an agent player participating in the SPD game with neighbors. At a given discrete moment, each cell can be in one of two states: either *C* or *D*. The state of a given cell will be considered as an action *C* (cooperate) or *D* (defect) of the corresponding player against an opponent from its neighborhood. We also assume that some *rule* (also called *strategy*) from the available set of rules is assigned to each agent–CA cell. Rules are used to change the cells’ states. The following set of six socially interpreted rules was considered in the SPD games: *all–C*: always cooperate. *all–D*: always defect. *k–D*: cooperate until not more than *k* neighbors defect; otherwise, defect. *k–C*: cooperate until not more than *k* neighbors cooperate; otherwise, defect. *k–DC*: defect until not more than *k* neighbors defect; otherwise cooperate. *p–C*: cooperate with probability $p_{C}$, where *k* is a user-predefined level of tolerance.

Each player playing a game with an opponent in a single round (iteration) receives a payoff according to the payoff function presented in Table 1. A received payoff is equal to *R*, *T*, *S* or *P*, where T>R>P>S. We assume that R=1, T=b , S=c, and P=a. We assume that players are rational and act in such a way as to maximize their payoff defined by the payoff function. To evaluate the level of collective behavior of the system, we will use an external criterion (not known for players) and ask whether it is possible to expect from the players selecting such actions $s_{ij}$, which will maximize the average total payoff (ATP) $\bar{u}\left(\right)$ (which corresponds to the maximization of the total number of cooperating agents) of the whole set of players:

(3) $$\bar{u}\left(s_{11},s_{12},\cdots ,s_{mn}\right)=\frac{1}{mn}\sum_{j=1}^{m}\sum_{i=1}^{n}\sum_{k=1}^{n_{ij}}u_{ij}\left(s_{ij},s_{i_{k}j_{k}}\right)/n_{ij},$$

where $n_{ij}$ is the number of opponents in the neighborhood.

Game theory predicts that the behavior of players is oriented toward achieving a Nash equilibrium (NE) point. We call the price of a NE a value of ATP at this point. The game can have many NE points with different ATP. The maximal ATP equal to *R* corresponds to selecting action *C* by all players. We will call this ATP the *maximal price point*; however, a solution corresponding to this point is not always a Nash point. To solve this problem, we introduce an *income sharing mechanism* [11] to the game, if necessary.

As the result of a single round of the game, each player participating in games with his neighbors collects some total cumulative payoff. The next phase of the round is a competition between players. The competition is based on a comparison of a cumulative payoff of a player with the cumulative payoffs of its neighbors. In the considered SPD game, we applied an evolutionary mechanism of a local tournament, where the winner of a competition was a player with locally the highest cumulative payoff. If a given player is a winner, it continues to use its rule to change its state, but in the opposite case its rule is replaced by a winner rule, and this new rule is used to change its state. CA which assume a possibility of changing their rules during an operation are called *second-order CA*. As a result of the competition, better rules are spread around the players, and worse-performing rules are eliminated. In the last phase of a single round, a strategy of a player can be mutated with some predefined probability, and next, the strategy is used to change the state of an agent cell. This new state is used in the next single game with opponents.

**Figure 2:**
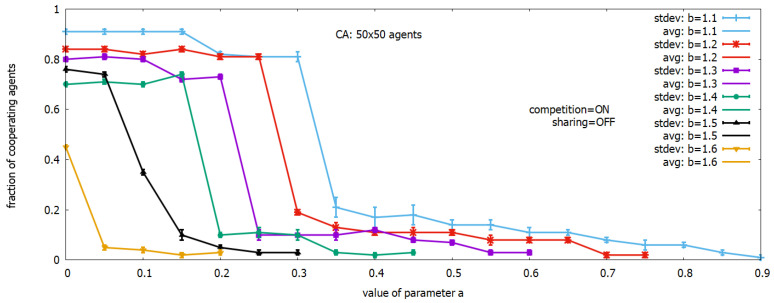
Level of cooperation of players in SPD games with 3 rules: *all–C*, *all–D*, and *k–D* as a function of parameters *a* and *b*.

**Figure 3:**
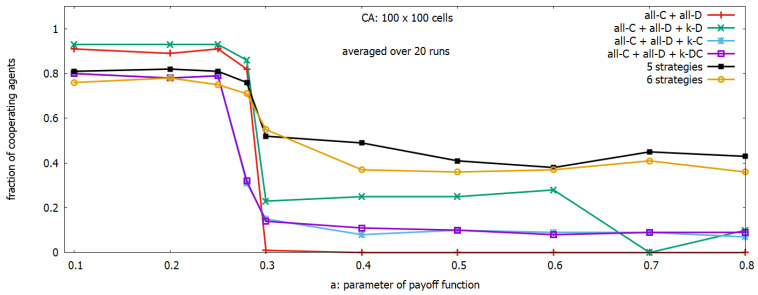
Fraction of cooperating agents with 6 different subsets of strategies as a function of the parameter *a*.

Figure 2 and Figure 3 show the results of some experiments demonstrating self-organizing futures of the SPD game model considered in this section. In the first experiment, it was assumed that to each CA cell, one of the following rules will be assigned: *all–C*, *all–D*, or *k–D*. Figure 2 shows how the level of cooperation in the multi-agent system depends on values *a* and *b* of the payoff function under a given set of three rules. In this experiment, a 2D spatial array of size 50×50 was used, and the presented results were averaged over 20 runs. The *income sharing mechanism* was turned off. The plots show the behavior of the multi-agent system for values of *b* ranging from 1.1 to 1.6, and for values of *a* changing between 0 and 0.9. One can see that under a given value of *b*, the fraction of cooperating agents strongly depends on values of *a*, but the highest level of players’ cooperation is observed for the lowest value of *b*.

Let us see the plot corresponding to parameter b=1.2 (in red). One can see that the highest level of cooperation close to 84% is achieved for a value of *a* in the range 0..0.25. It is a result of a small difference between the values *b* and R=1 (see payoff table). A difference not greater than 0.2 is too small to be attractive for a player to change its action from *C* into *D* and continue to play *D*. However, when the value of *a* increases, players that selected *D* will be more and more attracted by the NE point defined by the value of *a*, and returning to cooperation will be more difficult. Indeed, the number of cooperating agents decreases with the increase in the value of *a*. When a=0.3, the number of cooperating players suddenly drops to around 18% and continues decreasing when increasing the value of *a*. When a=0.7, the cooperation level is close to 0. When the value of *b* increases, the conditions for cooperation decrease faster, and for b=1.6 (in orange), some level of cooperation close to 0.45 is possible only when *a* is close to 0. One can conclude that a higher value of *b* means a higher payoff, enabling escaping from cooperation, which results in disrupting cooperation.

The purpose of the next experiment was to find out how different subsets of rules available from the set of basic rules influence the level of global collective behavior. It was assumed that b=1.2 in all experiments, a 2D spatial array of size 100×100 was used, and the presented results were averaged over 20 runs. Figure 3 shows the behavior of the multi-agent system when agents use subsets of rules. We considered 6 strategies corresponding to different subsets of rules: a subset {*all–C* + *all–D*} (plot in red), 3 subsets of strategies consisting of 3 rules: {*all–C* + *all–D* + *k–D*} (plot in green), {*all–C* + *all–D* + *k–C*} (plot in light blue), and  {*all–C* + *all–D* + *k–DC*} (plot in violet), a subset with 5 rules {*all–C* + *all–D* + *k–D* + *k–C* + *k–DC*} (plot in graphite), and a subset with all 6 rules (plot in orange).

We can see three similar regions of *a* for the behavior of the system, but we can also observe differences. The main differences for the region a<0.25 are the following: (a) the 3-rule subset containing the *k–D* rule (plot in green) is the best-performing one, (b) the remaining strategies composed of 3, 5 or 6 rules have good performance, and the 5-rule strategy set (plot in graphite) is the best performing among them, and (c) the performance of the 3-rule strategies with rules *k–C*, *k–DC* is much higher in comparison when they were used as single adaptive strategies. For a>0.3 we can observe (a) a similar performance of the system composed of 3 rules to that observed in experiments with single rules, and (b) a significant increase in the performance of the system composed of 5 or 6 rules, where the increase of the performance is the result of collective behavior caused by including rules *k–C* and *k–DC* to the pool, which were not efficient when working separately.

The results of the studies on SPD games conducted with the use of a second-order CA in the context of collective behavior of agent players have resulted [9] in developing a computational framework called the Competitive Co-evolutionary CA-based System, enabling the self-optimization of large distributed systems. The proposed approach has three components: (a) a multi-agent, social-like interpreted system, where the modeling of agents, discrete space and time is provided by 2-dimensional second-order CA, (b) an interaction of agents is described in terms of the SPD game, and (c) a local evolutionary mechanism of *competition*, based on the principle “adapt to the best neighbor”.

## 5. Multi-Agent System for WSN Coverage and Lifetime Optimization

An important step in interpreting the problem of WSN coverage and lifetime optimization in terms of a multi-agent system is the conversion of a WSN into the corresponding WSN graph. The conversion is based on the principle saying that two nodes of a WSN graph are connected if and only if they have at least one common PoI within their sensing range $R_{s}$ in a corresponding WSN. The number of neighbors of a given node depends on the value of $R_{s}$. Figure 1b shows a WSN graph corresponding to the WSN 12 from Figure 1a. We can see from Figure 1b that the nodes of the WSN graph correspond to the sensors of WSN 12. The nodes have a number of neighbors ranging from 2 to 6.

As we already mentioned, each node of a WSN graph is controlled by an agent $A_{i}$ of a multi-agent system consisting of *N* agents. Each agent has two alternative decisions (actions): $\alpha_{i}=C$ (battery is turned on) or $\alpha_{i}=D$ (battery is turned off). All agents will make discrete-time decisions regarding the activation of their batteries using certain rules (strategies) assigned to them, and a second-order CA will be used by agents. In this study, we applied a set of CA-based agent rules consisting of only two rules:

- *all–C*: always cooperate (turn on battery);
- *all–D*: always defect (turn off battery).

We will assume that the *i*-th agent (sensor) of the multi-agent system will take part in a variant of the SPD game related to the WSN coverage and lifetime optimization problem. It will be assumed that each agent knows the value of a global user-defined parameter, requested coverage $q_{r}$, and this value can be considered a local value, i.e., $q_{r}^{i}=q_{r}$. An agent will receive for his actions in the game some payoffs which depend on whether his current $q_{curr}^{i}$ is below or above the requested $q_{r}^{i}$, and he acts only on the base of his reward with the goal to maximize it.

The payoff function of a player participating in the SPD-like game defined by a WSN graph is given in Table 2. The payoff function assigns values to the *i*-th player in the following way:

(i) If he “turns off battery”, then he calculates his local value of coverage $q_{curr}^{i}$. If this value $q_{curr}^{i}\geq q_{r}^{i}$, then he receives reward *b* and otherwise, some punishment equal to *a*.
(ii) If he “turns on battery”, then he calculates what his value of $q_{curr}^{i}$ (denoted as $q_{curr}^{i-off}$) would be if in fact he would have “turned off” his battery. If $q_{curr}^{i-off}<q_{r}^{i}$, then he receives the reward equal to *d*, and otherwise, a penalty equal to *c*.

It is worth noticing that while the proposed payoff function presented in Table 2 was inspired by the payoff function from Table 1, in fact, it is different because it describes a specific application of the SPD game approach. In the SPD game presented in Section 4, each player was playing directly with each of his neighbor opponents. In our case, each agent-player related to a specific sensor does not play directly with each of his neighbors but with a virtual opponent representing a collective decision of a set of his neighbors. Therefore, while the assumption b>d>c>a concerning values of payoff function parameters for the SPD game was true, it not necessarily will be true for our game.

**Table 2:** Payoff function of SPD-like game to solve WSN coverage and lifetime optimization problem.

*i*-th Agent’s Action	Fulfillment of $q_{r}^{i}$	
C: turn on battery	$q_{curr}^{i-off}\geq q_{r}^{i}$	
	no	yes
	$\;\;\;\;\;rew_{i}^{on+}=d\;\;\;\;\;$	$\;\;\;\;\;rew_{i}^{on-}=c\;\;\;\;\;$
D: turn off battery	$q_{curr}^{i}\geq q_{r}^{i}$	
	no	yes
	$rew_{i}^{off-}=a$	$rew_{i}^{off+}=b$

**Figure 4:**
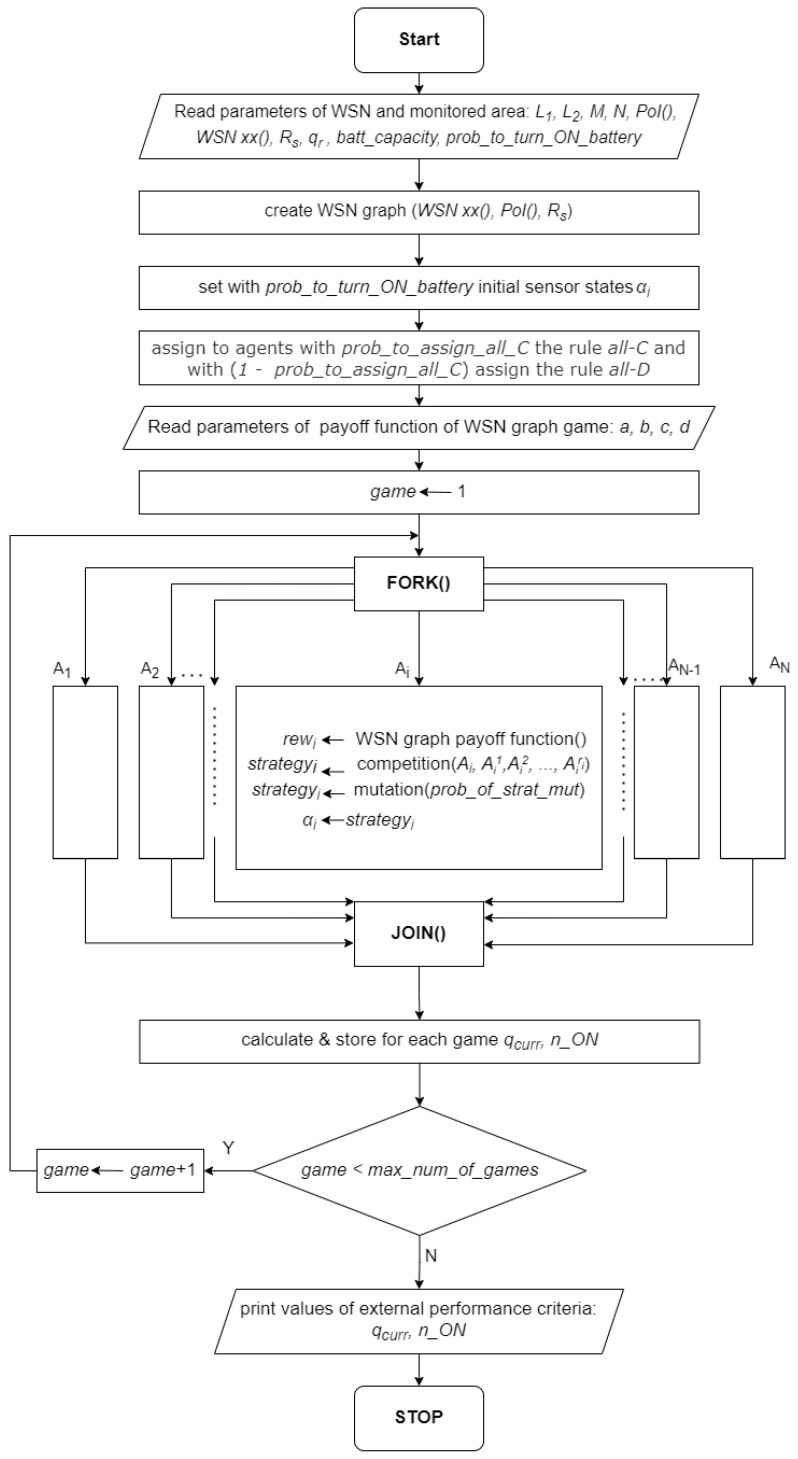
Flowchart of Competitive Co-evolutionary CA-based System for self-optimization of WSN.

The proposed approach to solving the coverage and lifetime optimization problem with the use of the SPD-like approach, providing self-optimization of WSN, is called *Competitive Co-evolutionary CA-based System*. Table 3 summarizes the parameters and variables used for a description of our approach. Figure 4 presents a flowchart of the proposed algorithm, and  Algorithm 1 presents its pseudocode.

The parameters used for the statement and a solution of the considered problem are presented in Table 3 and belong to one of four groups. The first group contains parameters describing the considered WSN and related to its monitored area. The second group represents the parameters of the multi-agent system created on the base of the WSN. The third group contains parameters of a WSN graph game, which is used in the distributed searching of a solution. The last group contains global parameters, which describe the goals of optimization. The crucial step for the considered self-optimization algorithm is to notice that the values of these parameters are external and not known for agents. Agents know only the values of their rewards and the rewards of neighbors and act in such a way as to maximize their personal rewards. During an iterated game consisting of max_num_of_games, it is expected that players will reach a Nash equilibrium, where further improvement of their personal rewards is not possible. Only external users having access to external performance parameters $q_{curr}$ and n_ON can see the values of these parameters at a NE point and judge the performance of the self-optimization.

Figure 4 presents details of the algorithm. We can recognize there are three parts: reading data and initialization, iterated game, and delivery of results. In the first part, after reading a WSN and the monitored area parameters, a WSN graph is created, which gives a possibility to interpret the WSN as a multi-agent system. The next two steps in the algorithm are (a) setting initial states $\alpha_{i}$ of batteries with a predefined probability prob_to_turn_on_batt, and (b) an initial assigning to the agents with a predefined prob_to_assign_all_C the strategy all–*C* and with (1 −prob_to_assign_all_C) the strategy all–*D*. This part ends with a reading of parameters a,b,c,d of a payoff function of the WSN graph game.
**Algorithm 1:** Solving by self-optimization the coverage and lifetime optimization problem.1:create a WSN graph and assign agents $A_{i}$ to the corresponding sensors $s_{i}$2:according to a predefined probability prob_to_turn_on_batt turn on the batteries of the sensors3:assign to agents according to a predefined probability prob_to_assign_all_C the rule (strategy) all–*C* and with (1 −prob_to_assign_all_C) the rule all–*D*4:**while** termination condition NOT TRUE **do**5:     **for all** agents $A_{i}$  **do in parallel**6:        an agent $A_{i}$ plays a game with his $r_{i}$ neighbors from the WSN graph and receives a payoff $rew_{i}$ according to the level of fulfillment of a local value of coverage $q_{r}$7:        an agent $A_{i}$ participates in a local competition with his $r_{i}$ neighbors which can result in replacing his strategy with a better-performing neighbor8:        according to a predefined probability of mutation prob_of_strat_mut, the rule of agent $A_{i}$ is modified9:        a battery state $\alpha_{i}$ is changed according to the rule currently assigned to the agent $A_{i}$10:     **end for**11:    calculate global performance characteristics of a multi-agent system-coverage $q_{curr}$ and a number n_ON of sensors turned on12:**end while**13:**Output 1:** the coverage $q_{curr}$14:**Output 2:** the number n_ON of sensors turned on

The second part of the algorithm describes the iterated game of players, which starts with setting the counter of games game to 1. The number of single games in the iterated game is defined by a user-predefined parameter max_num_of_games. In a single game, players $A_{1},A_{2},\cdots ,A_{i},\cdots ,A_{N}$ play their games with their neighbors at the same time in parallel, which is denoted by the pair of operators FORK() and JOIN(). In a single game, the $A_{i}$-th player performs the following steps: (a) he receives, according to the payoff function of the game, a reward $rew_{i}$ for his action $\alpha_{i}$ and the actions of his neighbors, (b) he takes part in an evolutionary competition with his neighbors, where he compares his $rew_{i}$ with the rewards of neighbors, which results in possibly replacing his $strategy_{i}$ by a strategy of a winner from the neighborhood, (c) his current strategy can be changed into the other one by a mutation which happens with a predefined probability prob_of_strat_mut, and (d) the current strategy assigned to him changes the state of his battery, setting in this way a new action $\alpha_{i}$ for the next game. After the completion of a single game, the values of external performance parameters $q_{curr}$ and n_ON are calculated and stored. The iterated game is continued until the game counter reaches the value max_num_of_games.

In the third part of the algorithm, after completing the iterated game, the values of the external performance parameters $q_{curr}$ and n_ON are printed, and the algorithm stops.

Algorithm 1 presents shortly a pseudocode of the Competitive Co-evolutionary CA-based System for the self-optimization of WSN.

## 6. Experimental Results

**Figure 5:**
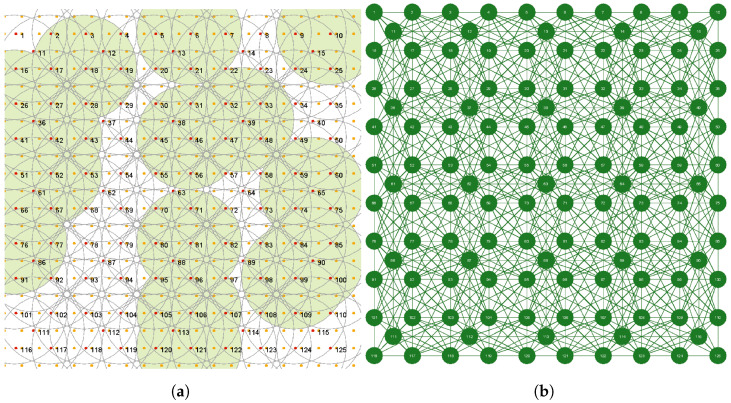
Instance of the problem: WSN 125 with 125 sensors, and $R_{s}=15$ m (**a**), corresponding WSN graph (**b**).

A number of simulation experiments have been conducted to learn the performance of the proposed methodology. An instance of the problem called WSN 125 consisting of 125 sensors with a sensing range $R_{s}=15$ m was used, and it was assumed that sensors are located in the monitored area (100 m × 100 m) with a number of PoI equal to M=441. Figure 5a presents the deployed WSN 125 with some number of sensors with batteries turned on (in green). The corresponding WSN graph of the multi-agent system is shown in Figure 5b.

It was assumed in all experiments that sensors are initially turned on with a probability equal to 0.5, and we expected the behavior of the multi-agent system providing the requested coverage of the monitored area $q_{r}$ with the use of a possibly minimal number of sensors, which are turned on. We assumed that the process of learning a solution lasts maximally 100 iterations, and also that in this process the batteries’ energy will not be consumed. The presented experimental results were averaged over 30 runs. The main purpose of the experiments was to find the optimal values for the parameters of the payoff function used by the players.

### 6.1. Searching of Optimal Parameter Value Settings for the Payoff Function

We started our experiments using the settings a=0,b=1.2,c=0.5,d=1, which fulfill the requested earlier stated relations for the SPD game (see, Section 4), having in mind that the final relations between these parameters should be found in the result of this study. We also assumed that the strategy mutation probability is equal to 0. We will call this set of values a *basic setting*. Searching for the optimal setting values of payoff function parameters was conducted under the assumption that the requested coverage of the monitored area $q_{r}=1$, i.e., that the whole 100% area should be monitored.

An important step of the algorithm is the participation of agents in local competitions with neighbors, resulting possibly in replacing their strategies with better-performing neighbor strategies. Our earlier experiments showed that competition based on the principle “adapt to the best neighbor” is not effective for irregular graph structures. We found out that the mechanism based on “a local proportional selection” (similar to the one used in genetic algorithms) performs well, and this mechanism is used in the competition.

The purpose of the first set of experiments was oriented on establishing optimal values for the payoff function parameters a,b,c,d. Generally, it is a relatively complex optimization problem that requires for an application some exact optimization method or some metaheuristic, but in this study, we will focus on establishing suboptimal payoff function parameters, providing a good behavior of the system. For this purpose, we will apply some sequence of steps that can be termed a hill climbing method, a very well-known simple optimization heuristic. The first step in this sequence is to find out how value changes of a given single payoff parameter influence values of the observed global objectives *q* and n_ON when the values of the remaining parameters are constant.

**Figure 6:**
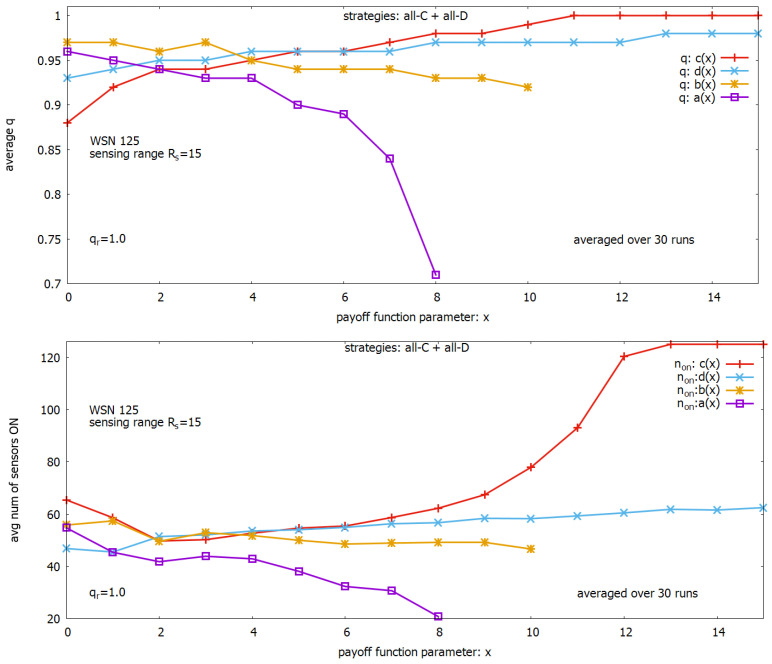
Influence of parameters of payoff function on: coverage *q* (**upper**), a number of sensors turned on (**lower**).

In this experiment, we allow in a given run to change only one of the four parameters, while the remaining parameters are set according to the basic settings. Ranges of changing the considered four parameters used in the experiments are different and are shown in Table 4. One can see that, for example, *a* changes in the range from 0 to 0.8, while *d* changes in the range from 0.5 to 2.0. To be able to observe the influence of all four payoff function parameters on changes of the global objectives *q* and n_ON within one plot (Figure 6 (upper) or (Figure 6 (lower)), we introduce an indexing parameter *x* which facilitates it. When we read from Table 4, e.g., x=6, we know from this table that it corresponds to an argument of either a=0.6 or b=1.6 or c=0.6 or d=1.1 used in Figure 6, and we can read from the plots the corresponding values of *q* and n_ON.

Analyzing the results of the experiments presented in Figure 6 showing the influence of parameters of the payoff function on global objectives *q* and n_ON, we can notice that the two parameters *a* (in violet) and *c* (in red) have a strong influence on the values of *q* and n_ON, while the influence of the two remaining parameters *b* (in orange) and *d* (in blue) is much lower. It is worth reminding that the desired behavior of the system in this experiment is providing full coverage of the monitored area (i.e., $q_{r}=1$) with the use of a possibly minimal number of sensors that are turned on. We can see (Figure 6 (upper)) that a=0 provides on average maximally q=0.96, and it requests turning on around 40 sensors on average (see, (Figure 6 (lower)). Increasing the value *a* strongly reduces *q*, which diverges further and further from the desired value. The parameter *c* acts in the opposite direction. When c=0, we observe the coverage q=0.88 and corresponding n_ON=65.4. When increasing the value *c*, we observe an increase in *q*, and starting from c=1.1 the value of *q* remains to be equal to 1. The requested number of sensors to be turned on initially decreases, and next starts to increase very fast. We can see that we have a multi-objective problem concerning two objectives *q* and n_ON, and we return to this issue later.

**Figure 7:**
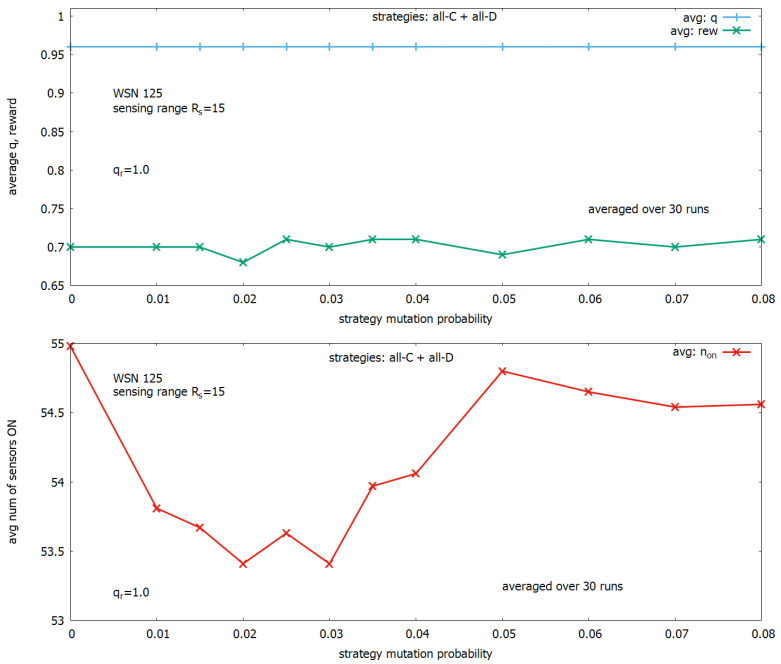
Searching for an optimal strategy mutation rate: influence of mutation rate on coverage *q* (**upper**), influence of mutation rate on a number of sensors turned on (**lower**).

To complete this phase of the study, we conducted the second set of experiments oriented on establishing an optimal value for the strategy mutation probability. The experiments were conducted under the basic settings of parameters, where only the strategy mutation probability was changed in the range from 0 to 0.08, and results are shown in Figure 7. One can see (Figure 7 (upper)) that under the considered range of changing the strategy mutation probability, the value *q* (in blue) remains on the same level equal to around 0.96. However, when we look at Figure 7 (lower), we notice that n_ON depends on the strategy mutation probability. On the basis of these results, we modify the basic settings of parameters by applying a strategy mutation probability equal to 0.02 and use these settings in the next experiments.

**Figure 8:**
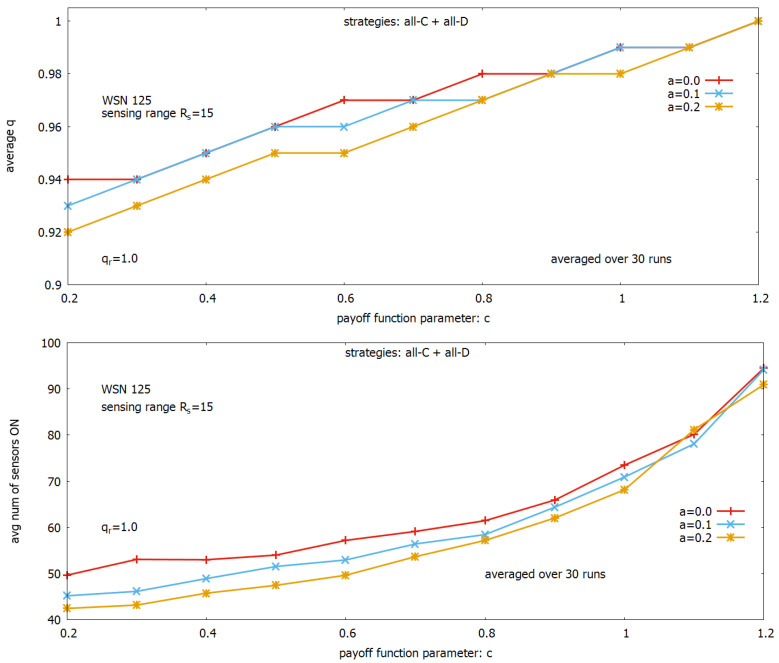
Searching of optimal values for the payoff function parameters *a* and *c*: influence of parameters on coverage *q* (**upper**), influence of parameters on the number of sensors turned on (**lower**).

To find out closer relations between the payoff function parameters *a* and *c* already noticed in Figure 6, we conducted a new set of experiments. Based on the results presented in Figure 6, we decided to limit our observation of the behavior of *a* to the range between 0 and 0.2 (below this range value of *q* becomes too low), and limit our observation of the behavior of *c* to 0.2≤c≤1.2 (below this value of *c* values of *q* are too low, and outside this value, n_ON becomes too large). The results of these experiments are presented in Figure 8 and show how values of *q* (see, Figure 8 (upper)) and n_ON (see, Figure 8 (lower)) depend on values of the parameter *c* and selected values of the parameter *a*.

Figure 8 (upper) shows that *q* depends on *c* near linearly for all considered values of *a*; however, a lower value of *a* provides a slightly higher coverage *q*. The whole range of changes of *q* is between 0.92 and 1, i.e., it is narrow. When we look at Figure 8 (lower), we can see that the cost of these relatively small changes of *q* expressed in a number of sensors turned on increases nearly exponentially. This cost depends on values of *a*: a higher value of *a* results in a lower value of n_ON. Based on these results, we can judge that for a given *a*, we have to do with pairs of solutions ((q(c),n_ON(c)), which form a Pareto front offering solutions with different quality of *q* and cost n_ON, giving a user the possibility to make a final decision.

**Figure 9:**
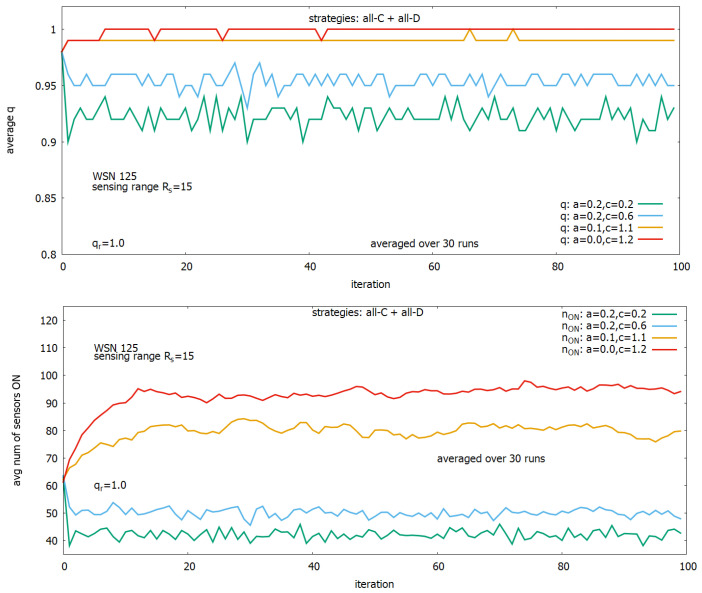
Solving of WSN coverage problem with second-order CA. Evolving in time steps of a final coverage (**upper**) and a corresponding number of active sensors (**lower**).

To better understand this idea, we show in Figure 9 runs of the system for four different pairs ((q(a,c),n_ON(a,c)). For a=0.2 and c=0.2, we can achieve a cover *q* equal to around 0.92 with a cost of around 43 sensors turned on (see, plots in green); for a=0.2 and c=0.6, we can have *q* equal to around 0.95 with the cost n_ON equal to around 50 (see, plots in blue); for a=0.1 and c=1.1 we can have *q* equal to around 0.99 with an increase of the cost n_ON to around 80 (see, plots in orange); and for a=0 and c=1.2, we can have *q* equal to 1 with large cost n_ON equal to around 95 (see, plots in red).

**Figure 10:**
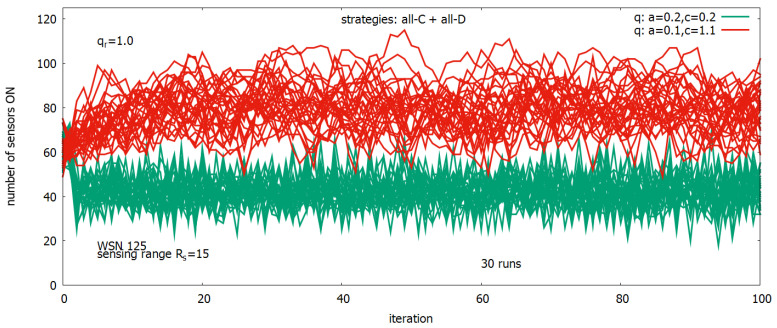
Thirty single runs for a=0.2, c=0.2 and (in red) and a=0.1,c=1.1 (in green).

Figure 10 shows details of two experiments presented in Figure 9 (lower). They each show single runs of the 30 runs (used for averaging of results), and they present changes in the number of sensors ON for a=0.2,c=0.2 (in red) and a=0.1,c=1.1 (in green). Comparing these results with the corresponding results from Figure 9 (upper), we can see that the highest value of *q* provided by the first setting is associated with a relatively large number of sensors, which are turned ON, and this process has also a relatively large standard deviation, while for the second setting, we observe a near twice lower number of sensors turned ON with a much lower standard deviation but providing only a slightly lower *q*.

Results of the conducted experiments until now give rise to judging that the already noticed four settings of the payoff function parameters are promising and worth further studies. Therefore, in the next subsection, we will study the behavior of the system under the four following settings: *setting 1*: a=0, b=1.2, c=1.2, d=1, *setting 2*: a=0.1, b=1.2, c=1.1, d=1, *setting 3*: a=0.2, b=1.2, c=0.6, d=1 and *setting 4*: a=0.2, b=1.2, c=0.2, d=1. These settings will be studied under a wide range of changing the user-defined $q_{r}$.

### 6.2. Solutions Offered by Self-Organizing System

**Figure 11:**
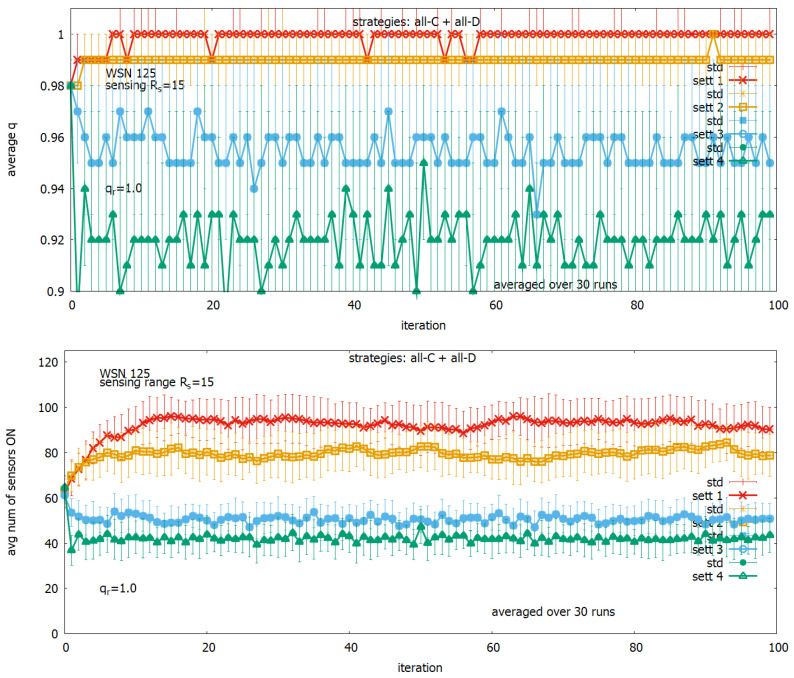
Solutions generated under *setting 1, setting 2, setting 3, setting 4* for $q_{r}=1$: coverage *q* (**upper**) and a corresponding number of active sensors (**lower**).

The purpose of the first experiment with the four selected settings was to find out how they influence the behavior of the system when the user-defined parameter $q_{r}$ is set to its maximum value $q_{r}=1$. The results of this experiment are shown in Figure 11. One can see that the near unconditionally fulfillment of the user-defined requirement takes place only for *setting 1* (see, in red, Figure 11 (upper)) and is related to exploiting the energy for a large number of sensors, which are turned ON ((see, Figure 11 (lower)). The remaining settings provide only a slightly lower value of *q* but with significantly lower consumption of the energy of sensors turned ON. The final decision concerning the selection of a given parameter setting for monitoring the area depends on the user’s requirement.

**Figure 12:**
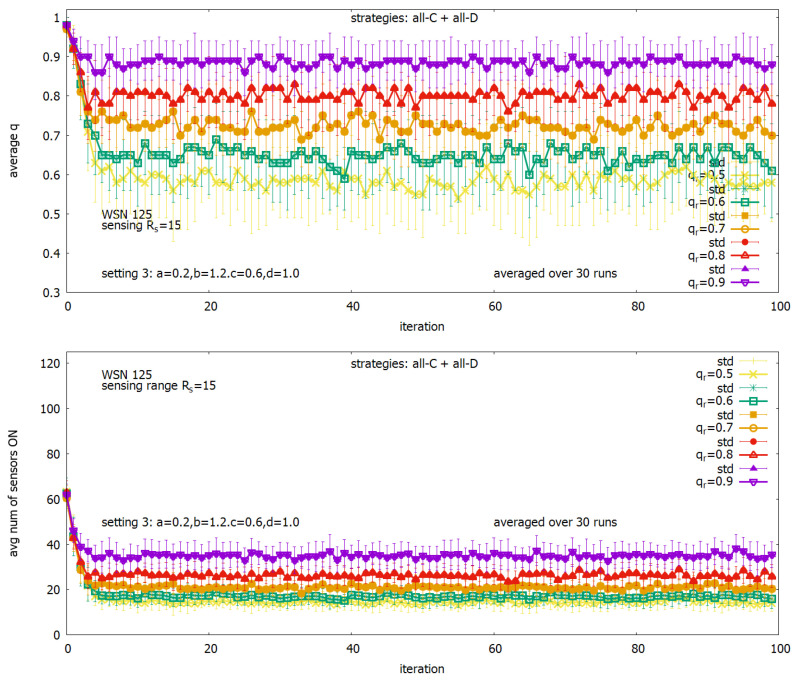
Solutions generated under *setting 3* for a range of $q_{r}$: coverage *q* (**upper**) and a corresponding number of active sensors (**lower**).

It is rather rarely requested to keep $q_{r}=1$ in a monitored system. Therefore, the next experiment is oriented toward checking how settings from the considered set of settings work in situations when requested $q_{r}<1$. We considered systems where requested $q_{r}$ was between 0.5 and 0.9. Results of the study show that only *setting 3* fulfills the requirements keeping the desired value $q_{r}$ by the system itself, and experimental results for this setting are presented in Figure 12. Figure 12 (upper) shows that the system near ideally keeps the required coverage for the whole considered range of $q_{r}$. Figure 12 (lower) shows that the required number of sensors being ON is relatively low.

**Figure 13:**
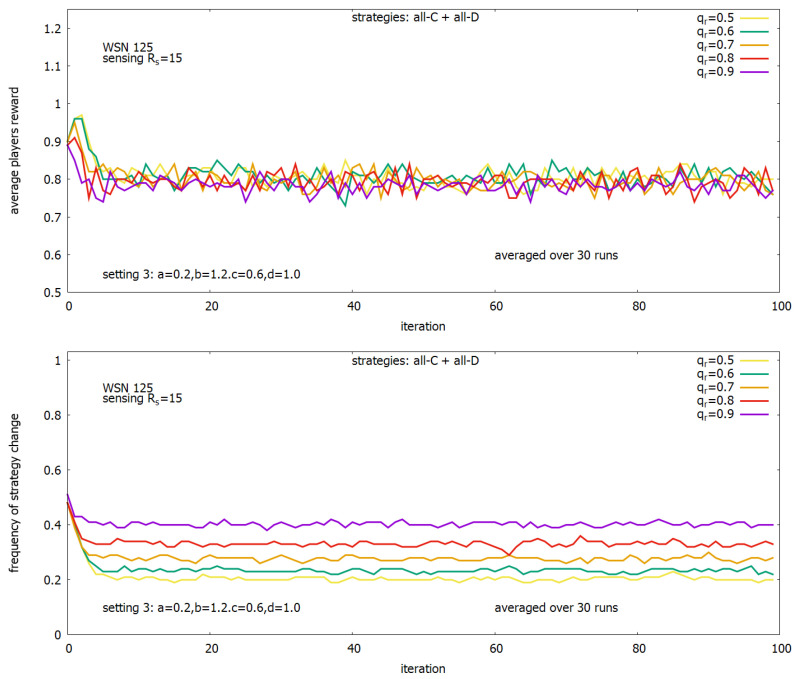
Solutions generated under *setting 3* for a range of $q_{r}$: average agents rewards (**upper**) and corresponding frequency of strategy changing (**lower**).

During the experiments, the other internal parameters of the system were also observed, and Figure 13 presents their behavior when *setting 3* was used. Figure 13 (upper) shows how the average total payoff of the agent players changed in time, and one can see that for the whole range of values of $q_{r}$, this value stabilizes after around 10 iterations and is close to 0.8 and practically does not depend on $q_{r}$. Figure 13 (lower) shows the behavior of another internal parameter: the frequency of strategy change during the game by players. One can see that the value of this parameter depends on $q_{r}$ and is higher for higher values of $q_{r}$ and keeps on some value in the range between around 0.2 and 0.4, depending on $q_{r}$.

The behavior of the system (not shown in the paper) with the use of the remaining 3 settings can be summarized in the following way. The system under *setting 1* and different values of $q_{r}$ behaves similarly to that shown in Figure 11 (upper). It keeps high values *q* ranging from 0.99 for $q_{r}=0.9$ to 0.97 for $q_{r}=0.5$ for the whole range of $q_{r}$. For the *setting 2*, the found values of *q* are also higher than requested, from only slightly higher for $q_{r}=0.9$ yielding q=0.97 to q = 0.88 for $q_{r}=0.5$. When the *setting 4* is used, the found solutions are slightly below the requested values of $q_{r}$, from q=0.83 for $q_{r}=0.9$ to q=0.58 for $q_{r}=0.5$.

## 7. Conclusions and Future Works

In this paper, we proposed a novel computational framework called *Competitive Co-evolutionary CA-based System*, allowing to design a parallel and distributed algorithm to solve the problem of coverage and lifetime optimization in WSN by self-optimization. To the best of our knowledge, it is the first successful work concerning developing self-organized WSNs. The main contributions of the paper are (a) developing a procedure for converting a WSN instance of into a WSN game graph enabling a multi-agent interpretation of the coverage and lifetime optimization, (b) applying a second-order CA as a novel class of reinforcement learning machines, which are able to learn CA rules in a variant of the iterated spatial PD game providing a Nash equilibrium-based solution, (c) working out a payoff function of the spatial PD game which associates the rewards of agents with the global WSN objectives, and (d) conducting a number of experiments showing that the system is able to self-optimize, i.e., achieve solutions corresponding to global optimization goals without direct knowledge about them. The proposed self-optimization system can be used in a real-time mode without the need for supervising by a central unit.

The proposed computational framework can be considered a general self-optimization evolutionary system consisting of three components: (a) a multi-agent system modeled by a 2-dimensional CA, (b) agents of the multi-agent system take place in a variant of the spatial PD game with a payoff function characterizing in some way a global goal of optimization and used locally by players, and (c) local evolutionary mechanisms of competition and mutation used to promote more successive CA rules working collectively on achieving a global goal without explicit knowledge of it. There exist some links between currently existing different variants of GA, such as a classical sequential GA, parallel GA (island and diffusion (called also cellular) models), co-evolutionary GA, and our model. We can point out two common features of the first three models. The first one is the existence of a population/sub-populations of individuals representing global solutions to the considered problem. The second one is that all individuals are evaluated with the use of a global function that is optimized and known for all individuals. In contrast, in our computational framework, while we also have a population of individuals represented by CA cells, each cell contains only a part of the solution. So, the algorithm evolves only one solution, the components of which are distributed among cells.

Looking at these models from the perspective of an algorithm classification presented in Section 2, we can notice that the first model—a classical sequential GA—is a centralized algorithm. Two parallel GA models—island and diffusion—belong to a class of distributed algorithms with different granularities of the agent’s representation, and a central agent is responsible for an evaluation of the fitness function of the individuals in terms of a global optimization function. Co-evolutionary GA models can also be recognized as distributed algorithms with the same features concerning the evaluation of the fitness function of the individuals. The model proposed in this paper can be classified as a self-optimization algorithm, where agents are fully distributed, i.e., they are autonomous in both making decisions and evaluation of their payoffs, which reflect in some way a global goal of the system.

The natural question is when and where the proposed framework can be applied. The answer is the following. It can be applied in systems that are considered large distributed systems and when the main issue is their self-optimization enabling their work in real time without external control. In a system that is relatively small and allows some level of centralization and collecting some global information about the state of the system, applying just common distributed algorithms discussed earlier will be more beneficial. In order to apply this approach for developing self-optimization algorithms , it is necessary to perform the following steps: (a) a given optimization problem should be converted into a multi-agent system, (b) a simple reinforcement learning machine should be used as an agent , and (c) a global optimization goal of the problem should be mapped into a payoff function of the game. The most important and difficult step is the translation of the global optimization goal into a payoff function of the players participating in the game. According to our knowledge, this issue in the area of multi-agent systems is open, and a potential solution may depend on the considered problem.

Our future work will be oriented toward better recognizing the future of the proposed computational framework. The first direction of the research will be oriented toward the continuation of the work presented in this paper and concerning the coverage and lifetime optimization of WSN. A number of issues seem to be interesting or important. In this paper, we used an elementary set of agent strategies consisting of two rules, although, as shown in Section 4, a set of three strategies may perform better than the elementary set. In our model, we also assume that all agents participate in a given single game, while a potential option is the existence of some level of asynchronicity when only a subset of the players participates in the game. An important issue that should be studied is the scalability of the algorithm, i.e., its performance for an increasing number of sensors and different instances of the problem. Other issues are further improving the payoff function and the analysis of its parameters from the point of view of the quality and stability of the solutions. The second direction of the research will be to extend the scope of the proposed framework to the development of self-optimization solutions for other optimization problems related to WSN and currently considered in the literature, and other problems related to computer-communication technologies, in particular, based on the Internet of Things and ambient intelligence.

## Figures and Tables

**Table 1:** Payoff function of a player in the SPD game.

Player’s Action	Opponent’s Action	
	Cooperate (C)	Defect (D)
Cooperate (C)	R=1	S=c
Defect (D)	T=b	P=a

**Table 3:** Parameters of Competitive Co-evolutionary CA-based system for self-optimization of WSN.

Parameters of WSN and Monitored Area	Description
$L_{1}$, $L_{2}$	sizes of a monitored area
$PoI(x_{k},y_{k})$	positions of points of interest in the monitored area
*M*	a number of PoI in the monitored area
*N*	a number of sensors deployed in the monitored area
WSNxx	a name of the considered WSN consisting of
	xx=N sensors
$R_{s}$	a sensing range of a sensor
*batt-capacity*	a battery capacity of a sensor
$q_{r}$	a requested coverage of the monitored area
prob_to_turn_on_batt	a probability of initial turning ON of a battery
**Parameters of a multi-agent system**	**Description**
WSNxxgraph	a graph of a multi-agent system corresponding
	to WSNxx with a given $R_{s}$ and PoI
$A_{i}$	an agent controlling a sensor $s_{i}$
$\alpha_{i}$	an action of $A_{i}$ changing a state of his battery:
	$\alpha_{i}=C$ (battery is turned ON),
	$\alpha_{i}=D$ (battery is turned OFF)
$strategy_{i}$	a strategy (CA rule) currently assigned to $A_{i}$
	from the set {all–*C*, all–*D*}
$A_{i}^{1},\;A_{i}^{2},\cdots ,A_{i}^{r_{i}}$	$r_{i}$ neighbors of $A_{i}$ in WSN graph
prob_to_assign_all_C	a probability of an initial assigning of the strategy
	all–*C* to a sensor
prob_of_strat_mut	a probability of a strategy mutation
**Parameters of WSN graph game**	**Description**
a,b,c,d	parameters of payoff function from Table 2
$rew_{i}$	a reward (payoff) of $A_{i}$ in a single game
max_num_of_games	maximal number of single games (iterations)
$rev_{i}^{on+},\;rev_{i}^{on-},$	rewards of $A_{i}$ depending on a situation
$rev_{i}^{off+},\;rev_{i}^{off-}$	in a local environment of a player
$q^{i}$	requested coverage for the area monitored by $A_{i}$
$q_{curr}^{i}$	a current coverage of the area monitored by $A_{i}$
**External performance criteria (not known for the multi-agent system)**	**Description**
$q_{curr}$	a current coverage of the monitored area
n_ON	a current number of sensors turned ON

**Table 4:** Ordering indexes *x* of arguments of payoff function parameters a,b,c,d.

x	0	1	2	3	4	5	6	7	8	9	10	11	12	13	14	15
*a*	0.0	0.1	0.2	0.3	0.4	0.5	0.6	0.7	0.8							
*b*	1.0	1.1	1.2	1.3	1.4	1.5	1.6	1.7	1.8	1.9	2.0					
*c*	0.0	0.1	0.2	0.3	0.4	0.5	0.6	0.7	0.8	0.9	1.0	1.1	1.2	1.3	1.4	1.5
*d*	0.5	0.6	0.7	0.8	0.9	1.0	1.1	1.2	1.3	1.4	1.5	1.6	1.7	1.8	1.9	2.0

## Data Availability

Data sharing not applicable.
